# Plant a Tree

**Published:** 1874-09

**Authors:** 


### PLANT A TREE.

The most luxuriant and the most rapid in its growth, thirty feet apart on each side of every street in every city, town, village, and public road in the nation, not only for the beauty of its appearance, not only for the comfort which the shade would afford to man and beast during the heats of summer, but for its great healthfulness. In many places in European countries, especially in Germany and Austria, there are seen miles and miles of rows of trees on either side of the public roads. One of the most beautiful of all country sights is that of a long avenue bordered by rows of overhanging trees.

A single tree, of a flourishing growth, evaporates about ten times as much water as falls during the summer over the space which the tree covers. All know that dry soils are healthy, and that wet soils are productive of a multitude of forms of fevers.

Observation has shown in a great number of places that a sickly locality has become healthy on its being covered with a growth of trees. Very recently a gentleman got the credit of being a natural born fool for purchasing all the ponds within a mile of his house. This occurred within an hour's ride of the City Hall, and within three years. He had built a house at a great expense for a country residence for his family, and found that the whole neighborhood suffered severely every autumn from various kinds of fevers. Making a use of his general readings, he attributed these autumnal maladies to the existence of these ponds; finding that there was no public spirit in the village, and not intelligence enough to discern that the stagnant ponds were the cause of the prevailing ailments, he determined to purchase the ponds himself and have supreme control over them; he then drained them in the winter; ploughed up their bottoms in the spring, sowed them with various kinds of grain and vegetable seeds, with the result that such prodigious crops were never known in all the region, with the other result, a most complete exemption from common intermittent and other autumnal diseases.

The streets of our cities and towns, if lined on either side with rows of luxuriant trees, would be disinfected of a large amount of the injurious gases which arise from the gutters, and from the various impurities which are found about them. Sulphuretted hydrogen has a peculiarly disagreeable odor, resembling that of a rotten egg, and yet every street in New York abounds with it in the summer time, as it is this which tarnishes the silver plated knobs of doors and bell pulls, even in Fifth Avenue. It is the luxuriant foliage of the country which aids in part to give life and purity to the air, and much more are trees needed in the town and along all our highways.

While it is true that luxuriant growths absorb pernicious gases, they make a house unhealthy, if it is embowered with trees, because dampness is promoted; hence, in passing through England, it is a rare thing to see trees near or in front of the country places of the gentry; even bushes are discarded, a beautiful, green lawn, fully open to the sun and air is almost universal. At the same time the end of a house of wood or stone is kept drier, yet cooler in summer, if it is covered with vines or trained branches of trees: drier because the leaves absorb the moisture; cooler because they ward off the sun's rays.

